# Comparative genomic analysis and mosquito larvicidal activity of four *Bacillus thuringiensis* serovar *israelensis* strains

**DOI:** 10.1038/s41598-020-60670-7

**Published:** 2020-03-26

**Authors:** Giselly B. Alves, Fernando L. Melo, Eugenio E. Oliveira, Khalid Haddi, Lara T. M. Costa, Marcelo L. Dias, Fabrício S. Campos, Eliseu J. G. Pereira, Roberto F. T. Corrêa, Sergio D. Ascêncio, Gil R. Santos, Guy Smagghe, Bergmann M. Ribeiro, Raimundo W. S. Aguiar

**Affiliations:** 1 Departamento de Biotecnologia, Universidade Federal de Tocantins, Gurupi, TO 77413-070 Brazil; 2 Departamento de Biologia Celular, Universidade de Brasília, Brasília, DF 70910-900 Brazil; 3 Departmento de Entomologia, Universidade Federal de Viçosa, Viçosa, MG 36570-900 Brazil; 4 Departamento de Entomologia, Universidade Federal de Lavras, Lavras, MG 37200-900 Brazil; 5 Rede de Biodiversidade e Biotecnologia da Amazônia Legal (Rede Bionorte), Universidade Federal do Tocantins, Palmas, TO 77413-070 Brazil; 6 Department of Plants and Crops, Ghent University, 9000 Ghent, Belgium

**Keywords:** Comparative genomics, Entomology

## Abstract

*Bacillus thuringiensis* serovar *israelensis* (*Bti*) is used to control insect vectors of human and animal diseases. In the present study, the toxicity of four strains of *Bti*, named T0124, T0131, T0137, and T0139, toward *Aedes aegypti* and *Culex quinquefasciatus* larvae was analyzed. The T0131 strain showed the highest larvicidal activity against *A. aegypti* (LC_50_ = 0.015 µg/ml) and *C. quinquefasciatus* larvae (LC_50_ = 0.035 µg/ml) when compared to the other strains. Furthermore, the genomic sequences of the four strains were obtained and compared. These *Bti* strains had chromosomes sizes of approximately 5.4 Mb with GC contents of ~35% and 5472–5477 putative coding regions. Three small plasmids (5.4, 6.8, and 7.6 kb) and three large plasmids (127, 235, and 359 kb) were found in the extrachromosomal content of all four strains. The SNP-based phylogeny revealed close relationship among isolates from this study and other *Bti* isolates, and SNPs analysis of the plasmids 127 kb did not reveal any mutations in δ-endotoxins genes. This newly acquired sequence data for these *Bti* strains may be useful in the search for novel insecticidal toxins to improve existing ones or develop new strategies for the biological control of important insect vectors of human and animal diseases.

## Introduction

During sporulation, the gram-positive bacterium *Bacillus thuringiensis* (*Bt*) produces crystalline inclusions consisting of δ-endotoxins (Cry or Cyt proteins) with insecticidal activity^1^. Genomic analysis has contributed to the identification of new genes coding for toxins that are active against different insect species including orders such as Lepidoptera, Diptera^2–7^, and Coleoptera^8^. Proteins with nematicidal^9–11^ and molluscicidal^12^ activities have also been described. In addition, genome sequencing of *Bt* strains with diverse ecological functions has been conducted, including a endophytic strain with potential utility in the biocontrol of phytopathogens^13^.

Sequencing of complete *Bt* genomes has allowed structural and functional analysis of new plasmids that enhance our knowledge of the pathogenic properties of *Bt* in targeting organisms^14–17^. One study reported the plasmid sequence of a *Bacillus thuringiensis* serovar *israelensis* (*Bti*) strain, and revealed that it may produce up to seven crystal-forming toxins, named Cry4A, Cry4B, Cry10A, Cry11A, Cyt1A, Cyt2Ba, and Cyt1Ca, which are all encoded by genes found in a single 127923 bp plasmid called pBtoxis^18^. The average size of the complete genome sequences of *Bti* is 6.1 Mb, with ~35% GC content of the chromosomal DNA and an average of 6132 coding sequences^19,20^. Genome sequences of seven *Bti* isolates have been reported so far^19,21–24^.

In this study, we sequenced the genomes of four *Bti* strains, specifically T0124, T0131, T0137, and T0139 that were collected from the soil of the Tocantins state in Brazil and determined their larvicidal activity against larvae of two important mosquito species of *A. aegypti* and *C. quinquefasciatus*. Then, to better characterize these strains, we performed comparative and phylogenetic analyses among their different genomes and compared the potential insecticidal toxin genes and other virulence factors of the four *Bti* strains with the commercial *Bti* strain H14. In case we identify high anti-mosquito activity with these strains, we believe these new data are useful in the continuous search for new insecticidal toxins to improve the existing ones or develop new strategies for the biological control of important insect vectors of human and animal diseases.

## Results

### Larvicidal activity and features of δ-endotoxins

**Figure 1:**
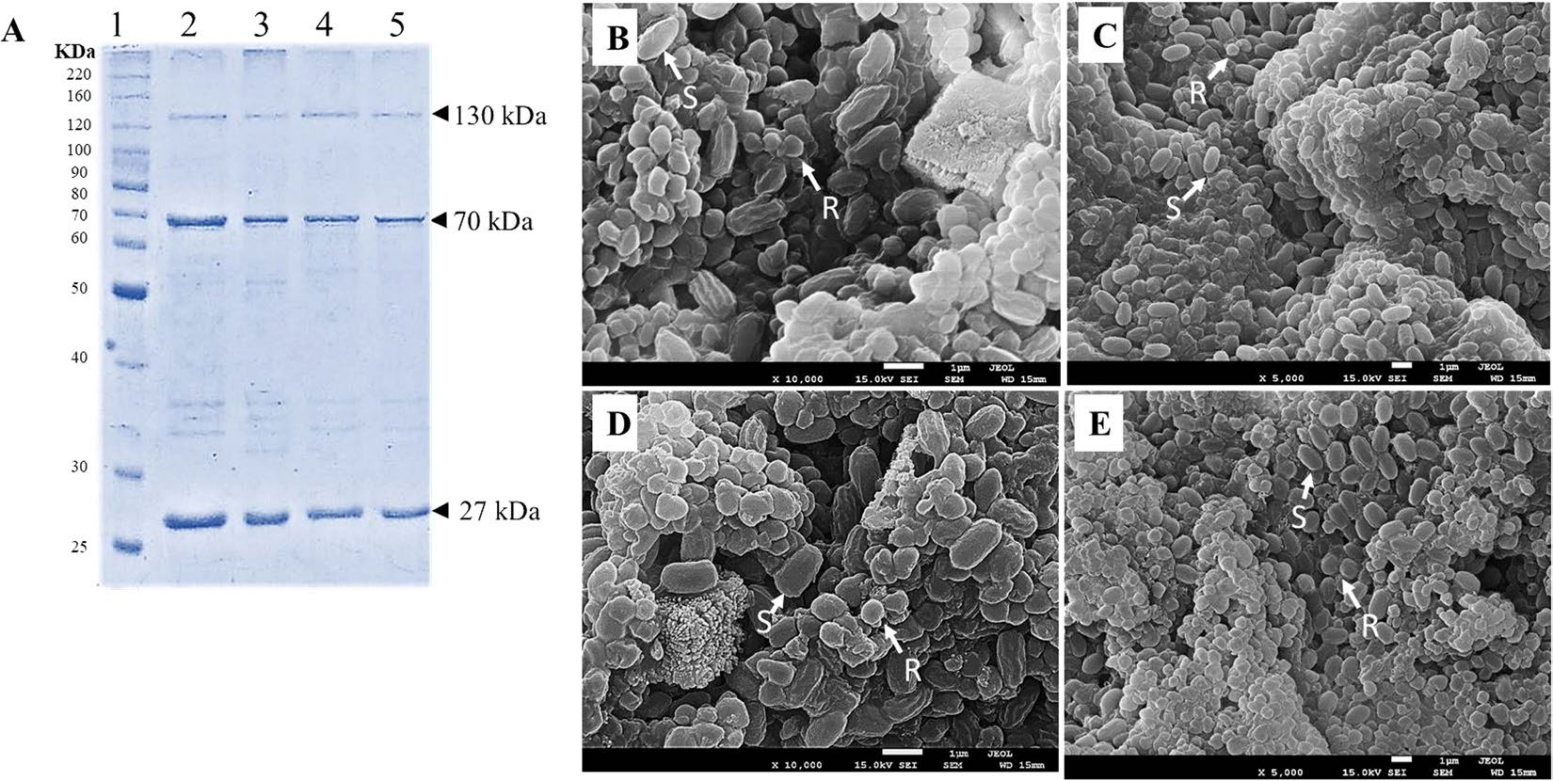
Crystal protein profile of *Bti* strains. Lane 1: molecular weight marker (Invitrogen); Lane 2: T0124; Lane 3: T0131; Lane 4: T0137; and Lane 5: T0139. Seven μg of solubilized crystals from each strain were analyzed by SDS-PAGE. The ultrastructural characterization of the spores and Cry proteins from T0124 (**B**), T0131 (**C**), T0137 (**D**), and T0139 (**E**) strains. All strains presented round crystals. Arrows indicate spores (S) and round crystals (R).

The spore-crystal mixtures of *Bti* strains T0124, T0131, T0137, and T0139 were tested against third instar larvae of *A. aegypti* and *C. quinquefasciatus*. The T0131 strain showed the highest larvicidal activity against *A. aegypti* (LC_50_ = 0.015 µg/ml) and *C. quinquefasciatus* (0.035 µg/ml) when compared to the other strains. Moreover, based on the toxicity ratios, the T0131 strain presented similar toxicity to the reference strain H14 (Toxicity ratio = 1.1 against *A. aegypti* and Toxicity ratio = 1.3 against *C. quinquefasciatus)* (Table 1). The T0124, T0137, and T0139 isolates showed lower toxicities compared to the H14 strain (Table 1). However, the SDS-PAGE analysis of crystal protein content revealed that all the strains have similar protein profiles. δ-endotoxins with molecular weights of 130, 70, and 27 kDa (Fig. 1A) and round morphology (Fig. 1B–E), characteristics of the *Bti* protein profile, were observed for the strains.

**Table 1:** Lethal concentrations of *Bti* strains to larvae of *A. aegypti* and *C. quinquefasciatus*, isolated in the town of Gurupi-TO, Brazil.

Insecticide type	Strains	No. of insects	LC_50_ (95% FI^a^) µg/ml	LC_95_ (95% FI^a^) µg/ml	TR^b^_50_ (95% CL)	*Χ*^2^	*P*
*A. aegypti*	T0124	525	0.069 (0.061–0.077)	0.243 (0.21–0.31)	5.2 (4.9–5.7)	4.36	0.36
	T0131	525	0.015 (0.012–0.018)	0.045 (0.03–0.07)	1.1 (0.9–1.3)	8.06	0.09
	T0137	525	0.165 (0.149–0.182)	0.534 (0.45–0.68)	12.6 (11.2–13.6)	5.06	0.28
	T0139	525	0.123 (0.096–0.157)	0.404 (0.28–0.79)	9.4 (8.2–10.3)	9.4	0.05
	H14	175	0.013 (0.011–0.016)	0.037 (0.03–0.05)	*	4.33	0.36
*C. quinquefasciatus*	T0124	450	0.172 (0.157–0.188)	0.467 (0.39–0.59)	6.4 (5.7–6.8)	1.97	0.58
	T0131	450	0.035 (0.031–0.039)	0.101 (0.08–0.14)	1.3 (1.2–1.4)	0.72	0.86
	T0137	525	0.239 (0.219–0.261)	0.630 (0.54–0.78)	8.6 (7.9–9.4)	7.07	0.13
	T0139	375	0.250 (0.220–0.283)	0.791 (0.63–1.10)	9.3 (8.2–9.8)	3.36	0.19
	H14	175	0.028 (0.024–0.032)	0.069 (0.06–0.10)	*	6.49	0.16

^a^FI = Fiducial Intervals; ^b^TR_50_ = Toxicity ratio determined by LC_50_ of given strain/LC_50_ of the reference strain H14 (*); 95% CL= 95% Confidence limits; *χ*^2^ = *Chi*-square for lack-of-fit to the probit model, and *P* = Probability associated with the *chi*-square statistic.

### Genome features

The average size of the chromosomal draft sequences of the T0124, T0131, T0137, and T0139 isolates was 5.4 Mb, with GC contents of ~35%. Chromosomes of these isolates contained 5477 (T0124), 5473 (T0131) and 5472 (T0137 and T0139) protein-coding genes. The number of tRNA genes was consistent over the strains (122) while small variation was seen in the number of rRNA (39–42) among them (Table 2).

**Table 2:** General features of the genome sequences of *Bti* T0124, T0131, T0137, and T0139 strains.

General features	T0124	T0131	T0137	T0139
Average coverage (n° reads)	10.2	10.3	17.5	11.0
Chromosome size (bp)	5 415 530	5 414 369	5 414 369	5 414 367
Sites no cover (%)	0.7	0.1	0.06	0.1
GC content (%)	35.2	35.3	35.3	35.3
CDS	5477	5473	5472	5472
tRNA	112	112	112	112
rRNA	39	42	42	42
Plasmids (n°)	6	6	6	6
Chromossome	1	1	1	1

**Figure 2:**
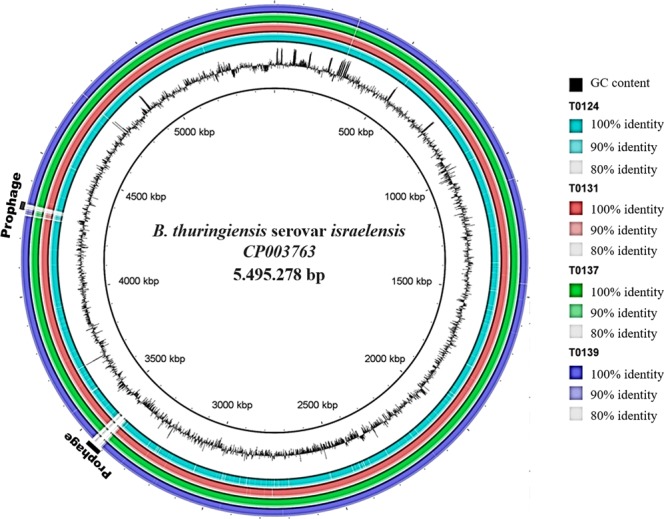
Comparative chromosomal nucleotide analysis of *Bti* strains. The concentric rings represent the sequences of T0124, T0131, T0137, and T0139 against the reference CP003763 strain. The black circle represents GC content of CP003763. The blue circle represents T0124, red circle represents T0131, green circle represents T0137, and purple circle represents T0139. Regions with less than 80% identity appear as blanks on each ring. This circular map was generated using the BLAST Ring Image Generator (BRIG) tool^48^.

Genome assembly showed high similarities in nucleotide identity with the HD-789 reference genome (Fig. 2). Regions (in blank) within positions 3 407 568 – 3 451 845 bp and 4 278 610 – 4 319 513 bp indicate two prophage sequences located on the reference chromosome which are absent in the T0124, T0131, T0137, and T0139 isolates (Fig. 2). All four strains contain six plasmids with average sizes of 5.4, 6.8, 7.6, 127, 235 and 359 kb. These replicons showed nucleotide identity greater than 99% with the extrachromosomal elements pTX14-1 (NC_002091), pTX14-2 (NC_004334), pTX14-3 (X56204), pBTHD789-2 (NC_018509), pBtoxis (NC_010076), and pHD1002-1 (NZ_CP009349), respectively (Table 3). The coverage for the plasmid assemblies was between 4000 and 14000 times for the 5.4, 6.8, and 7.6 kb plasmids and between 20 and 109 times for the 127, 235 and 359 kb plasmids.

**Table 3:** The nucleotide identity among plasmids of T0124, T0131, T0137, and T0139 strains and plasmids references.

Plasmids references	pTX14-1	pTX14-2	pTX14-3	pBTHD789-2	pBtoxis	pHD1002-1
Plasmids: Nucleotide identity (%)	pT0124-1: 99.8%	pT0124-2: 99.8%	pT0124-3: 99%	pT0124-4: 99.8%	pT0124-5: 99.9%	pT0124-6: 99.2%
	pT0131-1: 99.8%	pT0131-2: 99.8%	pT0131-3: 99%	pT0131-4: 99.8%	pT0131-5: 99.9%	pT0131-6: 99.2%
	pT0137-1: 99.8%	pT0137-2: 99.8%	pT0137-3: 99.5%	pT0137-4: 99.8%	pT0137-5: 99.9%	pT0137-6: 99.2%
	pT0139-1: 99.8%	pT0139-2: 99.8%	pT0139-3: 99.5%	pT0139-4: 99.8%	pT0139-5: 99.9%	pT0139-6: 99.2%

**Table 4:** General features of the assembly of complete plasmids of T0124, T0131, T0137 and T0139 strains.

Strains	Plasmids	Average coverage (n° reads)	Standard deviation	Plasmid Size (bp)	GC (%)	CDS	Access number
T0124	pT0124-1	14326	3312	5421	36.1	4	CP037884
	pT0124-2	15310	3005	6824	36	3	CP037885
	pT0124-3	5897	982	7697	35.3	9	CP037886
	pT0124-4	109	58.6	127922	32.4	117	CP037887
	pT0124-5	41.5	10	235425	36.6	242	CP037888
	pT0124-6	23.8	14	358206	32.3	338	CP037889
T0131	pT0131-1	14244	2742	5415	36.3	3	CP037453
	pT0131-2	14870	2408	6824	36	3	CP037454
	pT0131-3	5429	794.6	7697	35.3	9	CP037455
	pT0131-4	76.9	41.5	127923	32.4	117	CP037456
	pT0131-5	44.5	8.9	235425	36.6	241	CP037457
	pT0131-6	20.2	11	359437	32.3	336	CP037458
T0137	pT0137-1	8118	2540	5415	36.3	3	CP037459
	pT0137-2	8409	2676	6824	36	3	CP037460
	pT0137-3	3704	832	7697	35.3	9	CP037461
	pT0137-4	124.7	64.4	127923	32.4	117	CP037462
	pT0137-5	55.9	15.4	235425	36.6	241	CP037463
	pT0137-6	33.5	19.5	359440	32.3	336	CP037464
T0139	pT0139-1	4585	48.6	5415	36.3	3	CP037465
	pT0139-2	12014	3 013	6827	36	3	CP037466
	pT0139-3	4423	874.9	7697	35.3	9	CP037467
	pT0139-4	106	55.6	127930	32.3	117	CP037468
	pT0139-5	44	12.1	235425	36.6	241	CP037469
	pT0139-6	28	18.2	359438	32.3	336	CP037470

**Figure 3:**
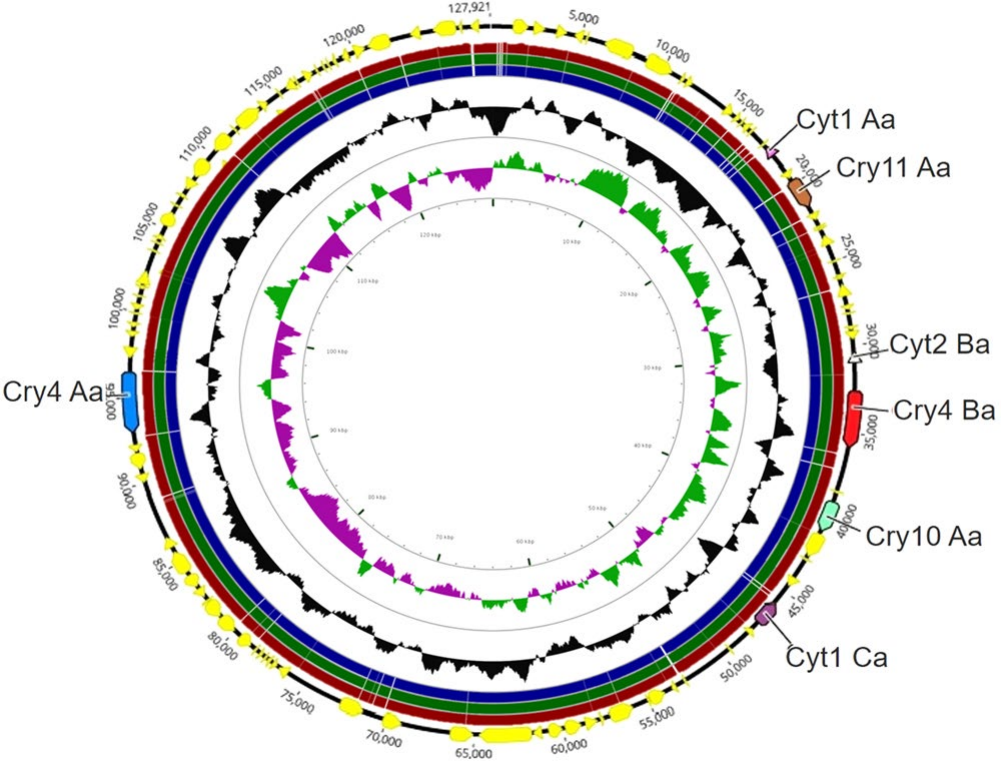
Comparative sequence map of pT0124-4, pT0131-4, pT0137-4 and pT0139-4 plasmids. The circles starting with the outermost ring are as follows: circle 1 (pT0124-4) showing the position of δ-endotoxins; circle 2 (pT0139-4), circle 3 (pT0137-4), and circle 4 (pT0131-4) show regions of sequence similarity representing darker regions detected by BLASTN in the primary sequence (pT0124-4). Circle 5 shows GC content (deviation from average) and circle 6 illustrates the GC skew in green (+) and purple (−). The circle with δ-endotoxins and the map was generated using the Geneious^47^ and CGView^56^ tool.

The 127 kb plasmid is the only one that encodes crystal-forming protein genes that are toxic to Diptera (*cry11Aa*, *cry4Aa*, *cry4Ba*, *cry10Aa*, *cyt1Aa*, *cyt1Ca*, and *cyt2Ba*) (Fig. 3) (Tables 3 and 4). No SNPs were found when the 127 kb plasmids of the different strains were compared.

### Comparative genomic analysis

**Figure 4:**
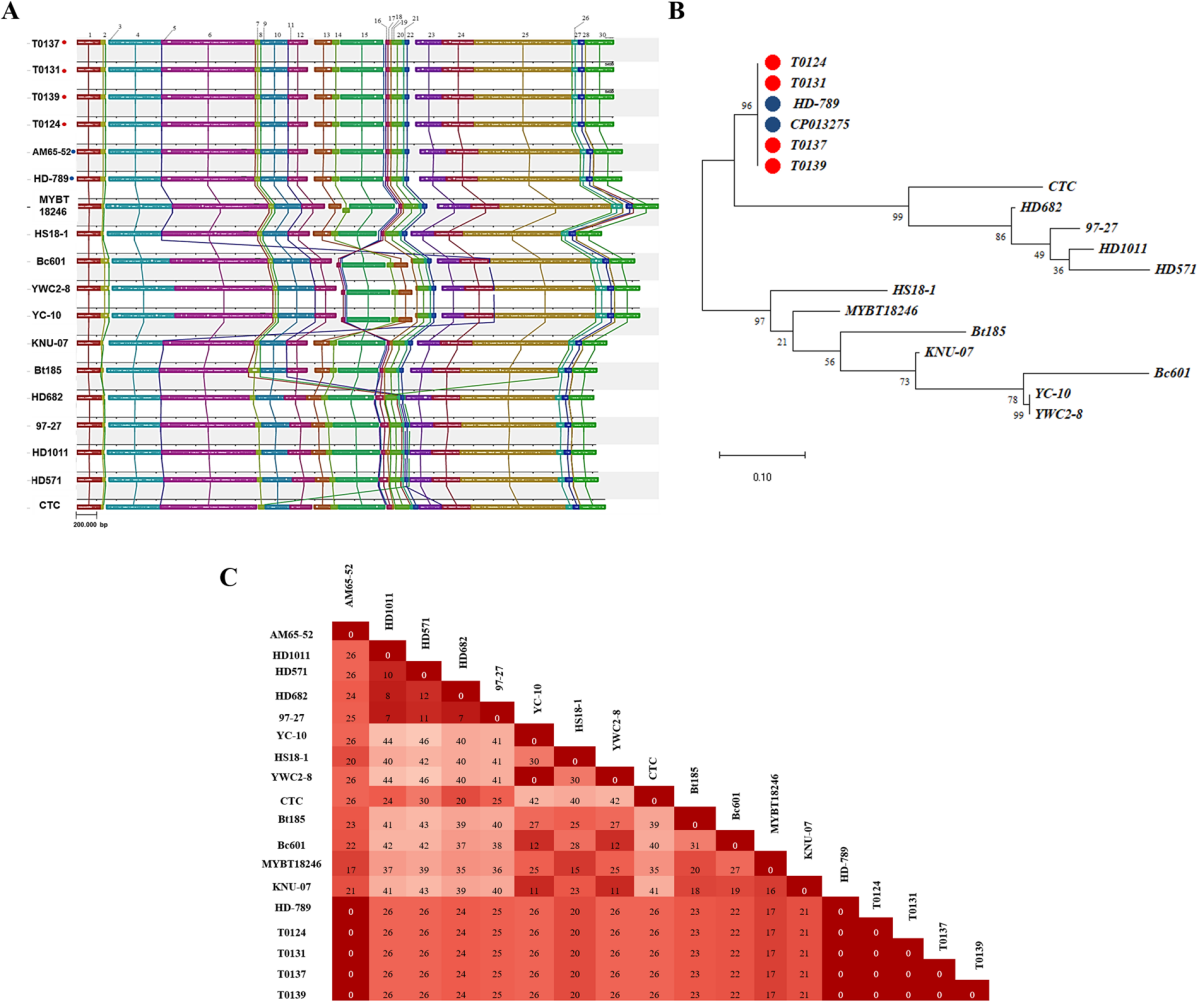
Comparative chromosome sequences of the isolates T0124, T0131, T0137, and T0139 with 14 genomes of other strains of *B. thuringiensis*. (**A**) Reciprocal LCBs in different sequences are indicated by the same colors and are connected by lines. (**B**) Phylogenetic tree based on the concatenated SNPs. The SNPs were called by CSI phylogeny 1.4^52^ using HD-789 strain as reference. The branch structure was confirmed by a bootstrap consensus tree inferred from 1,000 replicates in MEGA 10^53^. The scale bar indicates the evolutionary distance between the sequences determined by 0.10 substitutions per nucleotide at the variable positions. Red dots indicate the genomes of strains from the present study and blue dots indicate other genomes of *Bti* from the GenBank database. (**C**) The matrix shows the 2190 SNPs after pairwise comparison between isolates.

The genome drafts of the isolates T0124, T0131, T0137, and T0139 were compared to 14 other complete chromosomes of *B. thuringiensis* (Table 5) by phylogenetic analysis and Mauve alignment (Fig. 4). The Mauve alignment showed collinearity of genes among the isolates from this study and the *Bti* strains AM65-52 and HD-789, forming 32 locally collinear blocks (LCB) (Fig. 4A). The SNP-based phylogeny revealed close relationship between the isolates T0124, T0131, T0137, T0139 and the *Bti* strains AM65-52 and HD-789 (Fig. 4B). Although a total of 2190 SNPs positions were found in all analyzed chromosomes, no SNPs were found in the chromosomes of the isolates used in this study compared to *Bti* strains AM65-52 and HD-789 (Fig. 4C). Furthermore, the plasmids with 127 kb found in the four isolates (T0124, T0131, T0137, T0139) differed only by minor nucleotide changes (1 to 7 mutations) from the pBtoxis plasmid (NC_010076), and none of the nucleotide changes was related to the δ-endotoxins (Table 6).

**Table 5:** General features of chromosomes of *Bt* strains used in Mauve alignment and SNP-phylogenetic analysis.

Strain	Status of assembly	Chromosome size (bp)	GC (%)	CDS	Description	Access number	Reference
HS18-1	Complete	5 292 526	35.43	5234	Toxicity to Lepidoptera and Diptera	CP012099.1	Li *et al*.^58^
MYBT18246	Complete	6 752 490	35.4	6413	Toxicity to nematode	CP015350.1	Unpublished
YC-10	Complete	5 675 007	34.9	6028	Toxicity to nematode	CP011349.1	Cheng *et al*.^59^
YWC2-8	Complete	5 674 369	35.29	5692	Toxicity to Lepidoptera and Diptera	CP013055.1	Zhu *et al*.^60^
Bc601	Complete	5 627 121	35.30	5485	Used in fermentation for the production of vitamin C	CP015150.1	Jia *et al*.^61^
KNU-07	Complete	5 344 151	35.30	5111	Used in agriculture	CP016588.1	Unpublished
Bt185	Complete	5 243 635	35.30	4981	Toxicity to Lepidoptera	CP014282.1	Li *et al*.^62^
HD1011	Complete	5 232 696	35.5	5245	Medical relevance	CP009335.1	Johnson *et al*.^63^
HD682	Complete	5 213 295	35. 5	5201	Medical relevance	CP009720.1	Johnson *et al*.^63^
97-27	Complete	5 235 838	35.4	5216	Medical relevance	CP010088.1	Johnson *et al*.^63^
HD571	Complete	5 256 240	35.4	5219	Medical relevance	CP009600.1	Johnson *et al*.^63^
CTC	Complete	5 327 397	35.4	5268	High producer of S-layer protein	CP013274.1	Dong *et al*.^64^
HD-789	Complete	5 495 278	35.3	5551	Commercial insecticide isolate	CP003763.1	Dogget *et al*.^21^
AM65-52	Complete	5 499 731	35.0	5463	Toxicity to Diptera	CP013275.1	Bolotin, *et al*.^24^
T0124	Draft	5 415 530	35.2	5477	Toxicity to Diptera	CP037890	This study
T0131	Draft	5 414 369	35.3	5473	Toxicity to Diptera	CP035735	This study
T0137	Draft	5 414 369	35.3	5472	Toxicity to Diptera	CP035736	This study
T0139	Draft	5 414 367	35.3	5472	Toxicity to Diptera	CP035737	This study

**Table 6:** The SNPs of the pT0124-4, pT0131-4, pT0137-4, and pT0139-4 using pBtoxis (NC_010076) as reference.

Plasmids	Name	Position	Nucleotide Change	Amino Acid Change	Codon Change	Coverage	Polymorphism Type	Protein Effect	Variant Frequency	Variant P-Value (approximate)
pT0124-4	hypothetical protein CDS	99670	C -> T	S -> F	TCC -> TTC	408	SNP (transition)	Substitution	75.2%	0.0
	hypothetical protein CDS	99354	G -> A	H -> Y	CAT -> TAT	290	SNP (transition)	Substitution	76.9%	0.0
	hypothetical protein CDS	99343	A -> C		GGT -> GGG	304	SNP (transversion)	None	77.6%	7.6E-194
	hypothetical protein CDS	99255	AA -> CC	F -> G	TTT -> GGT	367	Substitution	Substitution	75.2%	0.0
	hypothetical protein CDS	99240	GA -> TG	S -> Q	TCG -> CAG	381	Substitution	Substitution	75.9%	0.0
	hypothetical protein CDS	99234	T -> G		AGG -> CGG	412	SNP (transversion)	None	76.7%	0.0
	hypothetical protein CDS	58097	G -> T		ACC -> ACA	241	SNP (transversion)	None	100.0%	7.9E-25
pT0131-4	hypothetical protein CDS	99670	C -> T	S -> F	TCC -> TTC	238	SNP (transition)	Substitution	79.8%	0.0
	hypothetical protein CDS	99282	T -> G	I -> L	ATA -> CTA	289	SNP (transversion)	Substitution	75.8%	0.0
	hypothetical protein CDS	99255	AA -> CC	F -> G	TTT -> GGT	274	Substitution	Substitution	78.1%	0.0
	hypothetical protein CDS	99240	GA -> TG	S -> Q	TCG -> CAG	282	Substitution	Substitution	78.4%	0.0
	hypothetical protein CDS	99234	T -> G		AGG -> CGG	312	SNP (transversion)	None	80.8%	0.0
	hypothetical protein CDS	99207	T -> C	I -> V	ATT -> GTT	299	SNP (transition)	Substitution	77.6%	0.0
	hypothetical protein CDS	58097	G -> T		ACC -> ACA	183	SNP (transversion)	None	100.0%	5.0E-19
pT0137-4	hypothetical protein CDS	58097	G -> T		ACC -> ACA	323	SNP (transversion)	None	99.7%	4.8E-63
pT0139-4	hypothetical protein CDS	99670	C -> T	S -> F	TCC -> TTC	302	SNP (transition)	Substitution	75.5%	0.0
	hypothetical protein CDS	58097	G -> T		ACC -> ACA	265	SNP (transversion)	None	100.0%	3.2E-27

**Figure 5:**
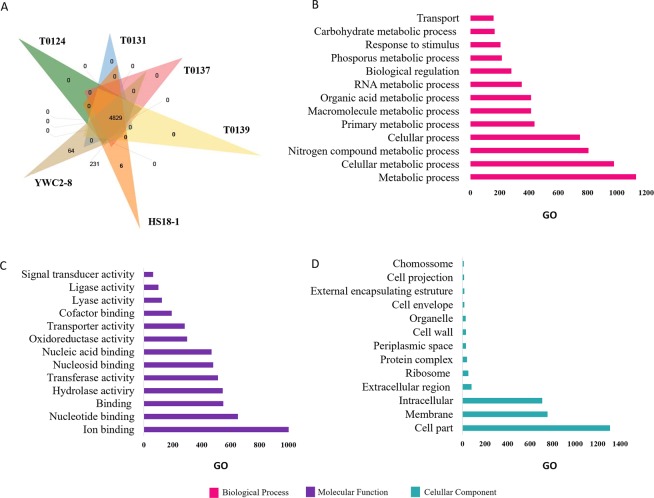
The Venn diagram of the strains from this study and other strains with toxicity to Diptera and summary of the functional gene ontology from shared OrthoVenn clusters. The Venn diagrams of T0124, T0131, T0137, T0139, HS18-1, and YWC2-8 (**A**). Summary of the functional gene ontology categories using GO slim^57^ for orthologous clusters in the Venn diagram overlapping regions are represented in the biological process (**B**), molecular function (**C**), and cellular component (**D**) categories.

A functional gene ontology analysis was performed among the four strains (T0124, T0131, T0137, T0139) and two strains of *B. thuringiensis* (HS18-1, and YWC2-8), which presented toxic bioactivity to dipteran insect and not associated to the serotype H14, followed by a summary from shared OrthoVenn clusters. The comparison of the inferred proteins among the strains of this study and the two other strains revealed 4 829 proteins shared by the strains and a total of 231 orthologous clusters shared by HS18-1 and YWC2-8 (Fig. 5A). The HS18-1 and YWC2-8 strains presented specific genes with 6 and 64 single clusters, respectively (Fig. 5A). The analysis of all Gene Ontology (GO) terms assigned to 4 829 orthologous clusters shared by the species showed 1 180 for metabolic processes, 1 001 for ion binding, and 1317 for cell parts (GO-inferred terms) (Fig. 5B–D).

**Figure 6:**
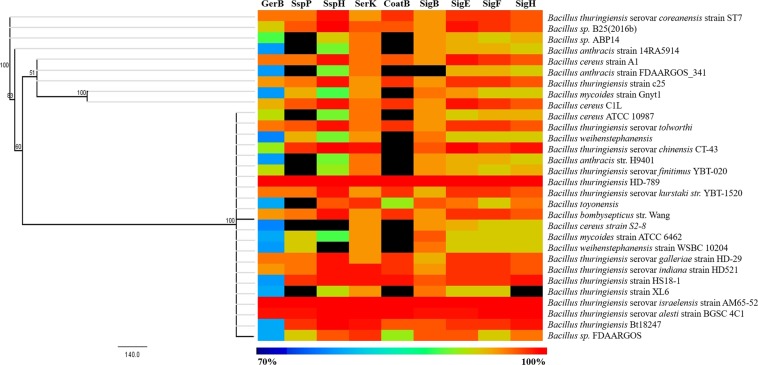
Heatmap comparison of the distribution of sporulation (GerB, SspP, SspH, SerK, CoatB) and sigma factor (SigB, SigE, SigF, and SigH) gene sequences among some species from the *Bacilli* group. Each column and line represents a gene and a Bacillus lineage, respectively, and percentage sequence identities between these species and the strains from this study were shown as colors ranging from 70% (dark blue) to 100% (red) as shown in the bottom. Undetected gene sequences are shown in black. The comparative analysis was performed using blastx and the Heatmap was generated using version 4.9.0 of the MeV tool^49^.

The important genes of the sporulation process previously described as variable and absent in some *Bacillus* species^25^ were analyzed. ORF sequences coding for the germination gene (GerB), small acid soluble proteins genes (SspP and SspH), sensor kinase (SerK) genes, coat gene of the spore (CoatB), and sigma factor genes (SigB, SigE, SigF, and SigH) were compared to the same genes present in other species of the *Bacilli* group (Fig. 6). Higher sequence identity was observed for *B. thuringiensis* HD-789 and *B. thuringiensis* serovar *israelensis* AM65-52. The GerB and SspH genes showed the highest sequence variability when compared with the sequences acquired in this study.

## Discussion

Here, the four new *Bti* strains T0124, T0131, T0137, and T0139, collected from the soil of the Tocantins state in Brazil, showed toxic activity to larvae of *A. aegypti* and *C. quinquefasciatus*. In addition to the fact that the mosquito strains were collected from locations not targeted by insecticide applications and hence presenting low risk of insecticide resistance build up. The *Bti* mode of action is distinct from neurotoxic or growth-regulating compounds used for mosquito control. These facts make *Bti* an effective alternative for controlling mosquito populations displaying or not resistance to these insecticides^26^.

The *Bti* strains analyzed in this study presented different lethal concentrations among them and when compared to a reference commercial strain of *Bti* (H14). However, the δ-endotoxin gene content and toxin protein profiles assessed by SDS-PAGE were very similar. The T0131 strain presented the highest toxicity for both insect vectors and, therefore, it is probably the most promising strain for biological control among the four isolated strains.

We performed whole genome sequencing of T0124, T0131, T0137, and T0139. However, differently from the toxicity results, the genomic analysis of these isolates indicated highly similar sequences. Previous genome comparison among strains of *Bt* revealed that 80% of the genes of this species are conserved, and the variability among *Bt* strains can be attributed to the acquisition of essential or non-essential genes from other microorganisms residing in the same microbial community^27^. In addition, *Bt* has an open pan-genome which is a characteristic of species that colonize different environments and have different genetic material exchange pathways^28^. *Bt* species comprise different subspecies and comparative analysis of the same subspecies may reveal genomically identical or highly related strains, even from different geographic regions. Such findings could be explained by the emergence of clonal lineages of pathogens that successfully colonized the biosphere, undergoing limited genetic exchange, thus representing homogeneous subspecies^23^. Similarly, studies have shown that *Bti* also present genomically similar strains, indicating the close relationship among them and suggesting a high degree of genomic conservation^29,30^ thus corroborating the results obtained in this study.

The assembled chromosomes of T0124, T0131, T0137, and T0139 did not show the presence of the two prophages present in the *Bti* reference HD-789^21^. Bolotin *et al*.^24^ also identified the sequences the two prophages in the *Bti* AM65-52 strain. Phage sequences, as well as plasmids, are said to be mobile genetic elements which also contribute to genetic diversity among species and are considered important tools in the divergence of strains and closely related bacterial species^31^.

Despite the presence of rearrangements and sequence inversions also that have been linked to the variability of genetically related species^32^, the strains from this study, HD-789 and AM65-52 showed collinear chromosomes. Although the polymorphism analysis indicated the presence of various SNPs in the *Bti* isolates, none of the mutations reported was related to the insecticidal activity^33^. The SNP-based phylogeny revealed close relation among the four isolates and other *Bti* isolates (HD-789 and AM65-52), in agreement with previous study^33^, reinforcing the close genetic relationship among these bacteria.

With regard to plasmids sequences, the high number of copies of extrachromosomal elements per chromosome can explain the high coverage of the plasmids with sizes of 5.4, 6.8, and 7.6 kb in this study^24,34^. Although some studies reported the ability of *Bti* strains to harbor up to nine plasmids, the assembly generated here revealed the presence of only six plasmids in the genomes of the isolates, which have also been reported elsewhere^21,24^. The 235 kb plasmids are presented in all sequenced genomes of these *Bti* strains. The 359 kb plasmid was described previously^24^ and is also found in the genomes of the T0124, T0131, T0137, and T0139 isolates.

Since the plasmids with 235 kb and 359 kb are not reported to show any crystal-forming proteins linked with toxic activity, they have not been described in this study. In fact, the plasmid with 235 kb has been described as a conjugative plasmid and the plasmid with 359 kb encodes various metabolite transporters^24^.

Since the 1980s, the direct relationship between plasmids and the pathogenicity of *Bt* was established, as they are responsible for carrying genes that express toxins active against target insects^35^. Plasmids of 127 kb were found in all isolates of *Bti* containing *cry* and *cyt* genes involved in insect toxicity. This type of plasmid, termed pBtoxis, is widely studied and described as the only plasmid capable of encoding the crystal-forming toxins of this bacterium^36^. In addition, pBtoxis also presents sequences with functions predicted to increase crystal formation and subsequent cell viability, acting as chaperones^32,36^. The additional cry4Ba coding gene in plasmid pBtoxis-like has been reported to account for the increased effectiveness in mosquito larvae killing of *Bti* strain^33^. In the present study, SNPs analysis of the plasmids carrying the genes encoding the mosquitocidal endotoxins did not reveal any mutation in pT0131-4 what could explain the high toxicity of T0131 strain.

Different strains of *Bt* within the same serotype may share a highly related plasmid pattern; this relationship is most evident in different strains of *Bti* H14 serotype, which, although isolated from different geographic origins, have the same basic plasmid pattern, sometimes even identical^37^. Therefore, our results, that show a high degree of genomic conservation among the strains T0124, T0131, T0137, and T0139, are consistent with previous studies.

The functional gene ontology analysis from shared clusters showed a unique set of proteins identified only in the genome of the YWC2-8 isolate associated with magnesium transport and in the HS18-1 isolate associated with vitamin B6 catabolic processes and pyridoxal 4-dehydrogenase activity (Fig. 5A). The summary of the functional gene ontology showed diversity for metabolic process category (Fig. 5B). The metabolic processes play important roles in the insecticidal activity of *Bt* because metabolic pathways are regulated to provide amino acid, carbon, and energy substances for sporulation and massive synthesis of crystal toxins^38,39^.

Heatmap analysis shows that sporulation genes and sigma factors are conserved among *Bti* strains, while the SspH and GerB gene showed highest variability. Although spore formation is central to the definition of *Bacilli*, these genes have been described as variable and absent in some species as a result of niche-specific constraints that may lead to variability in the detection of stress conditions, spore resistance, and germination^25^.

The comparative analysis of four new genomes of *Bti* carried out in the present study revealed their very high identity of nucleotide sequence. Furthermore, the results presented here are important for evolutionary studies of this species and potentially may contribute to the improvement of existing strategies or the development of new approaches in biological control that use these bacteria. Further investigations aiming to evaluate potential differences at transcriptomic/proteomic levels during specific phases (e.g., middle vegetative, early sporulation and late sporulation) of the four *Bti* strains will contribute to clarify the higher larvicidal activity described here for the T0131 strain.

## Methods

### Isolation of Bti strains

*B. thuringiensis* serovar *israelensis* (serotypes H14) T0124, T0131, T0137, and T0139 strains were isolated from a soil sample collected in Tocantins state (Brazil) according to the previously described protocol^40^. The bacterial strains were cultured at 28 °C for 12 h using the streak plate method on Luria-Bertani (LB) solid medium (10 gL^−1^ tryptone, 5 gL^−1^ yeast extract, 10 gL^−1^ NaCl, and 20 gL^−1^ Agar). Single bacterial colonies of each strain were inoculated in the LB liquid medium at 28 °C with shaking for 16 h.

### Spore-crystal protein preparation and crystal analysis by SDS–PAGE

Spore-crystal mixtures were obtained according to the protocol described previously^41^. For SDS-PAGE analysis, the crystals were purified using hexane and low speed centrifugation according to the previously described method^42^. Proteins were suspended in a small volume of phosphate-buffered saline (136 mM NaCl, 1.4 mM KH_2_PO_4_, 2.6 mM KCl, 8 mM Na_2_HPO_4_, and 4.2 ml H_2_O; pH 7.4), and fractionated by electrophoresis on 12% SDS-PAGE gels^43^.

### Scanning electron microscopy

The characterization of the spores and *Cry* proteins from the T0124, T0131, T0137, and T0139 strains was performed by scanning electron microscopy. The strains were cultivated in NYSM agar medium at 30 °C for 72 h, then a loop of the isolate was collected and diluted in sterile water. A volume of 100 µL of this dilution was deposited over metallic supports and dried for 24 h at 37 °C, covered with gold for 180 s using an Emitech apparatus (model K550; Quorum Technologies, Lewes, UK), and observed under a Zeiss scanning electron microscope (model DSM 962; Carl Zeiss AG, Oberkochen, Germany) at 10 or 20 Kv.

### Mosquitoes and toxicity bioassays

The colonies of *A. aegypti* and *C. quinquefasciatus* were established from insects collected from the field in regions of transition between the urban and rural areas in the state of Tocantins, Brazil, (11°40′55.7″ latitude S, 49°04′3.9″ longitude W), where no insecticides have been used for the control of mosquitoes. The insects were maintained in the Entomology Laboratory of the Federal University of Tocantins, Gurupi Campus, according to the methodology described previously^44^. Adult mosquitoes were maintained on a 10% aqueous sucrose solution and the blood of live Wistar rats (*Rattus norvegicus albinus*). The larvae were reared in plastic containers (35 cm × 5 cm) and were fed a sterilized diet (80/20 mix of chick chow powder/yeast). All bioassays were conducted at 26 ± 1 °C, 60.0 ± 5% RH, with a 12 h light-dark photoperiod. All applicable international, national, and institutional guidelines for the care and use of animals were followed. Bioassays were conducted using the suspension isolated from the spore-crystal mixtures against third instar *A. aegypti* and *C. quinquefasciatus* larvae. The concentrations were determined as described previously^45^. Seven concentrations were used for each spore-crystal mixture from each strain. Sterile distilled water was used as a control, and the larval mortality was recorded 24 h post inoculation. Three replicates with 25 larvae for each spore-crystal mixture were performed for all tested concentrations and for the non-treated control group. The spore-crystal mixture from the H14 strain was used as a reference.

### Whole genome sequencing, assembly, and annotation

Total genomic DNA was extracted and purified using a Wizard® Genomic DNA Purification Kit (Promega, Madison, WI, USA) according to the manufacturer’s instructions. DNA concentration and purity were measured using a NanoDrop™ 8000 (Thermo Fisher Scientific, Waltham, MA, USA). Whole genome sequencing was performed on the Illumina MiSeq™ platform using a paired-end application (2 × 150 bp) (Illumina, San Diego, CA, USA). The read quality of the sequenced libraries was analyzed using FastQC software v 0.11.3^46^ and sequence reads were trimmed to yield a minimum Phred quality score > 20. The genome assembly was performed using Geneious v 10.1.3^47^. The *de novo* assembly was performance using Geneious assembler with medium sensitivity settings and allowing contigs with matching ends to circularize. The linear contigs were extended. For this, the reads were mapped back to the linear contigs and the resulting contigs were used as seeds for another attempted assembly until no further extension. Finally, the extended linear contigs were aligned and reordered using as reference the genome *Bti* HD-789 (accession number CP003763) from the “map to reference” tool with minimum overlap identity of 85%. The circular contigs were used to investigate plasmid-like sequences, by matching them against plasmid bank with custom BLAST tool. Genome annotation was added by the NCBI Prokaryotic Annotation.

### Comparative genomic and phylogenetic analysis

Comparative chromosome-sequences analysis among T0124, T0131, T0137, T0139 and reference HD-789 was performed using BRIG (BLAST Ring Image Generator)^48^. The comparative analysis of the gene sequence of sporulation for the strains considered in this study and other species form the *Bacilli* group was performed using blastx, and the heatmaps were generated using the MeV tool version 4.9^49^. Venn diagrams generation and orthologous cluster annotation for biological process, molecular function, and cellular component categories were achieved using OrthoVenn^50^. The collinearity and phylogenetic analysis among T0124, T0131, T0137, and T0139 and others 14 chromosomes of *Bt* was performed. The collinear analysis and display of results were conducted using Mauve with the parameters reported previously^51^. The phylogenetic tree based on single nucleotide polymorphisms (SNPs) was performed by CSI phylogeny 1.4 web^52^ using the default parameters and HD-789 as reference. The SNPs were named, concatenated and aligned, and the tree was constructed using the maximum likelihood method. The phylogeny tree inferred was reviewed using MEGA X software^53^ with 1000 replicates. The pBtoxis (NC_010076) was used as reference for the SNPs analysis of pT0124-4, pT0131-4, pT0137-4, and pT0139-4 using Geneious v 10.1.3^47^, “Find SNPs/InDels” tool, with minimum coverage of 10, minimum variance frequence 0.75.

### Nucleotide sequence accession number

The Whole Genome Shotgun projects of PRJNA521267, PRJNA521275, PRJNA521276, and PRJNA521307 Bti strains were deposited in DDBJ/ENA/GenBank under the accession numbers CP037890, CP035735, CP035736, and CP035737.

### Statistical analyses

Concentration–mortality curves were estimated via probit analysis using the PROBIT procedure in the SAS statistical software package^54^. The differential susceptibility among mosquito species to H14 and the T0124, T0131, T0137, and T0139 *Bti* strains was assessed based on the estimated LC_50_ (i.e., the lethal concentration capable of killing 50% of tested mosquito species) of each strain and the toxicity ratios (TR_50_) were estimated by dividing the LC_50_ value obtained for the T0124, T0131, T0137, and T0139 *Bti* strains by the LC_50_ value obtained for the H14 standard strain^55^. The 95% confidence intervals estimated for these toxicity rates were considered to be significantly different if they did not include the value 1^55^.

### Ethical approval

All applicable international, national, and institutional guidelines for the care and use of animals were considered in the present investigation.

### Informed consent

All the authors of this manuscript accepted that the paper is submitted for publication in the *Scientific Reports* journal, and report that this paper has not been published or accepted for publication in another journal, and it is not under consideration at another journal.

## Supplementary information

Suplementary Information.
