# Supplementary material for: Comparative genomic analysis and mosquito larvicidal activity of four Bacillus thuringiensis serovar israelensis strains
Source: Sci Rep. 2020 Mar 26;10:5518. doi: 10.1038/s41598-020-60670-7 (PMC7099026; doi:10.1038/s41598-020-60670-7)

Supplementary Information

# Comparative genomic analysis and mosquito larvicidal activity of four *Bacillus thuringiensis* serovar *israelensis* strains

Giselly B. Alves[1], Fernando L. Melo[2]. Eugenio. Eduardo de Oliveira[3], Khalid Haddi[3], Lara T. M. Costa[1], Marcelo L. Dias[1], Fabrício. S. Campos[1], Eliseu J. G. Pereira[3], Roberto F. T. Corrêa[1], Sergio D. Ascêncio[4], Gil R. Santos[1], Guy Smagghe[5], Bergmann M. Ribeiro[2], Raimundo W. S. Aguiar[1]*

[1]*Departamento de Biotecnologia, Universidade Federal de Tocantins, Gurupi, TO, 77413-070, Brazil.*

[2]*Departamento de Biologia Celular, Universidade de Brasília, Brasília, DF 70910-900, Brazil.*

[3]*Departmento de Entomologia, Universidade Federal de Viçosa, Viçosa, MG, 36570-900, Brazil*

[4]*Rede de Biodiversidade e Biotecnologia da Amazônia Legal (Rede Bionorte), Universidade Federal do Tocantins, Palmas, TO, 77413-070, Brazil.*

[5]*Department of Plants and Crops, Ghent University, 9000 Ghent, Belgium.*

**Supplementary Figure 1.** Full-length gels (original) used to build the Figure 1 in the manuscript Alves et al. 2019.

(*A*) Original gel

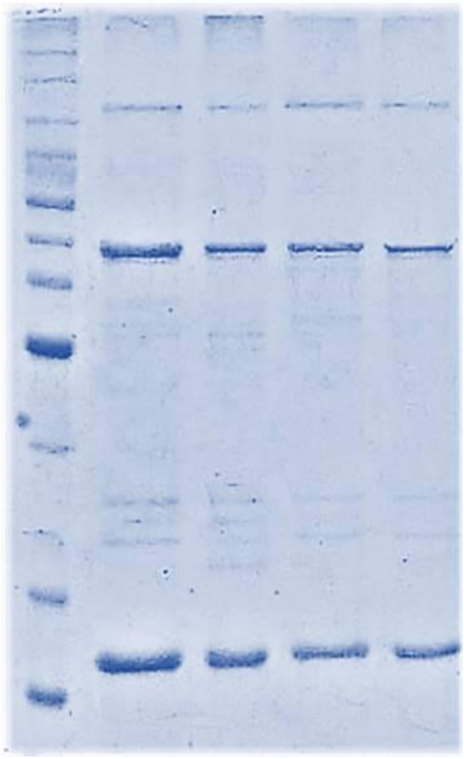

(*B*) Original gel with the legends (see **Fig 1*A***)

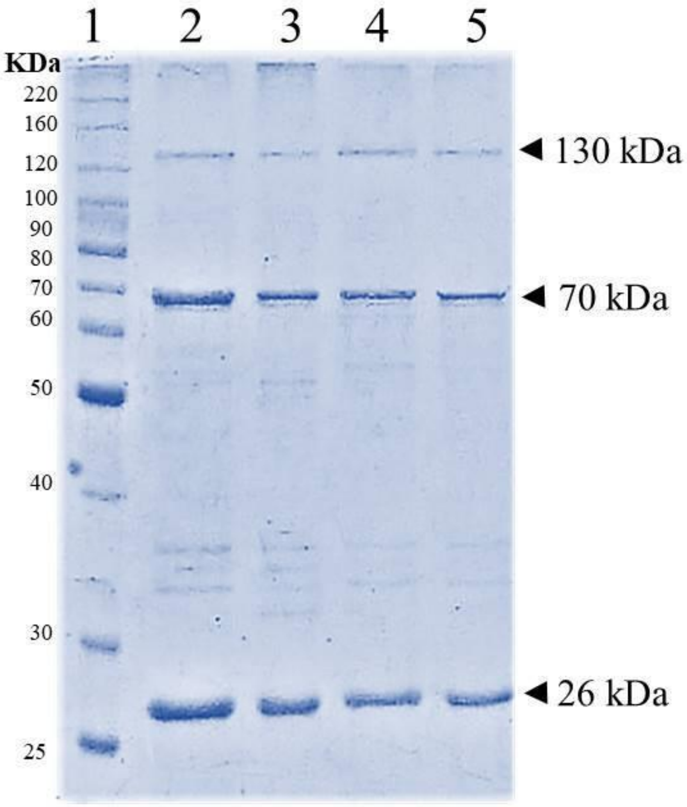

Supplement: Supplementary file 1 — Suplementary Information. [file 41598_2020_60670_MOESM1_ESM.pdf]
